# Endoplasmic Reticulum Stress May Play a Pivotal Role in Lipid Metabolic Disorders in a Novel Mouse Model of Subclinical Hypothyroidism

**DOI:** 10.1038/srep31381

**Published:** 2016-08-19

**Authors:** Lingyan Zhou, Shuyan Ding, Yujie Li, Laicheng Wang, Wenbin Chen, Tao Bo, Kunpeng Wu, Congcong Li, Xiaojing Liu, Jiajun Zhao, Chao Xu, Ling Gao

**Affiliations:** 1 Department of Endocrinology and Metabolism, Shandong Provincial Hospital affiliated to Shandong University, Jinan, Shandong, 250021, China; 2 Institute of Endocrinology, Shandong Academy of Clinical Medicine, Jinan, Shandong, 250021, China; 3 Shandong Clinical Medical Center of Endocrinology and Metabolism, Jinan, Shandong, 250021, China; 4 Experimental Animal Center, Shandong Provincial Hospital affiliated to Shandong University, Jinan, Shandong, 250021, China; 5 Scientific Center, Shandong Provincial Hospital affiliated to Shandong University, Jinan, Shandong, 250021, China; 6 Jinan central hospital affiliated to Shandong University, Jinan, Shandong, 250021, China

## Abstract

Subclinical hypothyroidism (SCH) is becoming a global health problem due to its increasing prevalence and potential deleterious effects. However, the molecular mechanisms underlying the lipid metabolic disorders in SCH have not been fully clarified. Additionally, progress in elucidating the exact pathogenesis of SCH has been hampered by the lack of optimized mouse models. Methimazole (MMI) was applied to construct a noninvasive SCH mouse model. Eight-week-old C57BL/6 mice were administrated MMI through the drinking water. After 12 weeks, the MMI-treated mice showed the diagnostic criteria for SCH: increased serum thyrotropin (TSH) levels with constant thyroid hormone levels that persisted for approximately 8 weeks. Notably, SCH mice presented evident lipid metabolic disturbances, including dyslipidemia and hepatic lipid accumulation. Further analysis showed that hepatic endoplasmic reticulum stress (ER stress) was induced in the SCH mice or by the elevation of TSH *in vitro*, likely via the IRE1α/XBP-1 pathway. Interestingly, when we used 4-phenyl butyric acid to repress ER stress in SCH mice for 4 weeks, dyslipidemia and hepatic lipid accumulation were both significantly alleviated. Our findings indicate that an optimized SCH mouse model could be established using MMI, and ER stress may play a pivotal role in the lipid metabolic abnormalities in SCH.

As stated in *The Lancet*, “The world faces a burden of thyroid disease that has reached epidemic proportions”^1^. In particular, subclinical hypothyroidism (SCH), the most common thyroid dysfunction, has drawn intensive interest due to its increasing prevalence and potential deleterious effects. Biochemically, SCH is defined as high serum thyrotropin (thyroid stimulating hormone, TSH) levels, while free thyroxine (FT_4_) is within normal levels^2^. In adults, the prevalence of SCH ranges from 4% to 20% of the population in different regions^3^,^4^,^5^. The prevalence of this condition is increasing, from 4.3% in 1988 to 9.0% in 1995 in the United States^4^,^6^ and from 11.3% in 2003 to 19.3% in 2012 in India^7^,^8^. SCH, which is often accompanied by lipid metabolic disorders, is an independent risk factor for atherosclerosis [OR (odds ratio) = 1.9], myocardial infarction (OR = 3.1), and nonalcoholic fatty liver disease (NAFLD) (hazard ratio = 2.21)^9^,^10^. Thus, there is a need for further research on SCH, and a suitable SCH animal model will be conductive to investigating the pathological characteristics of this disease. However, there is still a lack of optimal models for research.

Both hemi-thyroid electrocauterisation^11^ and thyroidectomy^12^ have been used to establish SCH animal models. Nevertheless, there are some inadequacies in these two methods: traumatic processes could trigger inevitable damage to animals, and both methods required researchers skilled in operation. Both of these methods are based on physical damage to the mouse thyroid, which is too small to be precisely manipulated. Could antithyroid drugs, such as methimazole (MMI), be used to construct a noninvasive, simple and ideal SCH mouse model? To date, correlation studies have not been performed.

SCH is often concomitant with lipid metabolic abnormalities^13^,^14^,^15^, which may be the primary cause of its deleterious effects. Our previous findings have revealed some of the mechanisms underlying lipid metabolism disorders in SCH^16^,^17^. However, whether the endoplasmic reticulum, the specialized organelle of lipid biosynthesis, is involved in the dyslipidemia accompanying SCH is unknown. Currently, there have been no relevant reports. The signaling pathways triggered by endoplasmic reticulum stress (ER stress) can initiate or exacerbate lipotoxicity, inflammation, insulin resistance and apoptosis, which are all key factors in causing dyslipidemia-related diseases^18^. This prompted us to investigate whether ER stress plays an important role in the lipid metabolic disorders of SCH.

In this study, a novel SCH mouse model was constructed using MMI, and the whole body metabolic status of the SCH mice was evaluated using metabolic chambers. We further demonstrated that ER stress may play an important role in the dyslipidemia of SCH for the first time. Our findings provide a feasible and simple SCH animal model, which laid the foundation for further research on SCH. Moreover, we determined the molecular mechanism underlying the lipid metabolic disorders in SCH and provided a new intervention target for its prevention and treatment.

## Results

### Development of a novel SCH mouse model was successfully constructed

**Figure 1:**
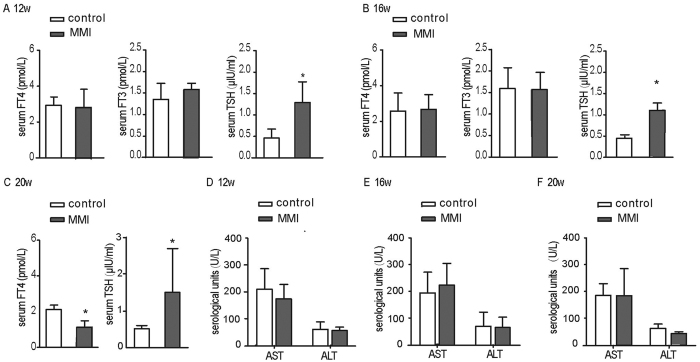
Development of a novel SCH mouse model was successfully constructed. Male C57/BL6 mice were administered methimazole (MMI, 0.08 mg/kg·BW·d, MMI-treated group, n = 6–8) in drinking water or a corresponding volume of vehicle (control group, n = 6–8) for 12 weeks, 16 weeks or 20 weeks. **(A)** The serum FT_4_, FT_3_ and TSH levels were assayed in the 12th week. **(B)** The serum FT_4_, FT_3_ and TSH levels were assayed in the 16th week. **(C)** The serum FT_4_ and TSH levels were assayed in the 20th week. **(D)** The serum ALT and AST levels were assayed in the 12th week. **(E**) The serum ALT and AST levels were assayed in the 16th week. **(F)** The serum ALT and AST levels were assayed in the 20th week. The results are expressed as the mean ± SD. *p < 0.05 compared with the control group.

The hallmark of SCH is elevated serum TSH and normal FT_4_ levels. Hence, we continuously monitored the variation in serum TSH and thyroid hormone levels after administering MMI to the mice in the drinking water. After treatment with MMI (0.08 mg/kg·BW·d) for 12 weeks, the mice showed the diagnostic criteria of SCH compared to the controls. Serum TSH levels in the MMI-treated group significantly increased, and serum thyroid hormone (FT_3_ and FT_4_) levels were indistinguishable between the treated and control groups (Fig. 1A). When MMI was administered in their drinking water for 16 weeks, the SCH state persisted (Fig. 1B). However, after 20 weeks of treatment, serum FT_4_ levels were decreased, and serum TSH levels were still elevated in the MMI-treated mice compared to the controls (Fig. 1C), which indicated that the model mice had entered the phase of clinical hypothyroidism (CH). These results suggested that after the application of MMI, mice indeed progressed from SCH to CH, and the SCH state persisted for nearly eight weeks.

Meanwhile, it was necessary to evaluate the safety of MMI. Therefore, serum ALT and AST levels, which are indicators of liver function, were monitored. As shown in Fig. 1D–F, the serum ALT and AST levels in MMI-treated mice were similar to those observed under normal physiological conditions in control mice from the 12th week to the 20th week. Therefore, the potential effect of MMI at the present dose on hepatic function could be excluded.

### The whole body metabolic status of the SCH mouse model

**Figure 2:**
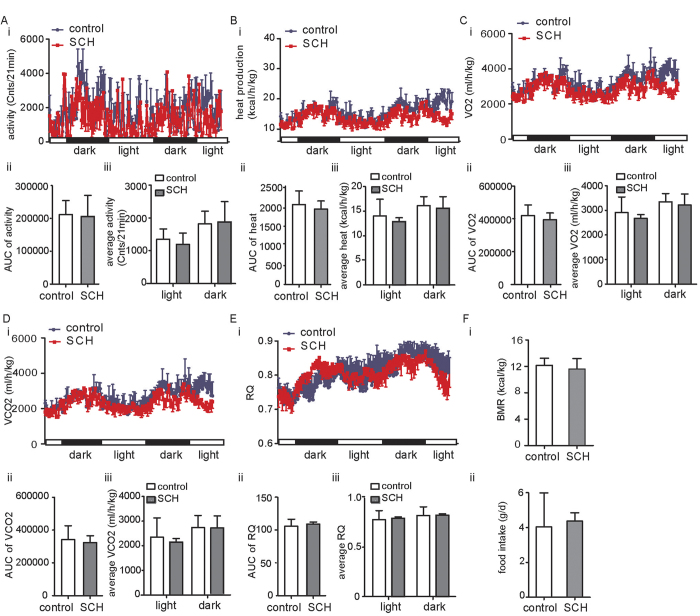
The whole body metabolic status of the SCH mouse model. Male C57BL/6J mice at 8 weeks of age were randomly assigned to groups (n = 6–8). Mice were allowed ad libitum access to food and different types of water. Vehicle or MMI (0.08 mg/kg/d) was administrated to the mice. After 16 weeks of treatment, the mice (n = 3) were placed into metabolic chambers to measure oxygen consumption, CO_2_ production, total activity, and other parameters. The data are presented as the mean ± SD. *p < 0.05 compared with the control group. **(A)** i, the total physical activity was recorded. ii, the area under the curve (AUC) for physical activity was displayed. iii, the average physical activity for each group was calculated in the light cycle and the dark cycle, respectively. **(B)** i, the heat production was recorded. ii, AUC for heat production was displayed. iii, the average heat production for each group was calculated in the light cycle and the dark cycle, respectively. **(C)** i, the VO_2_ (volume of oxygen consumed) of different groups was recorded. ii, AUC for VO2 was displayed. iii, the average VO2 for each group was calculated in the light cycle and the dark cycle, respectively. **(D)** i, the VCO_2_ (production of carbon dioxide) of different groups was recorded. ii, AUC for VCO2 was displayed. iii, the average VCO2 for each group was calculated in the light cycle and the dark cycle, respectively. **(E)** i, the respiratory quotient (RQ) of different groups was recorded. ii, the AUC for RQ was displayed. iii, the average RQ for each group was calculated in the light cycle and the dark cycle, respectively. **(F)**The BMR (basal metabolic rate) and food intake were recorded. The data are presented as the mean ± SD.

Generally, there are distinct clinical symptoms in CH patients, such as sluggish actions or motion, depressed whole body metabolism and reduced heat production. In contrast to CH, these symptoms are rarely observed in SCH. To comprehensively assess the SCH mouse model, we used metabolic chambers to evaluate the whole body metabolic status when MMI was administered in drinking water for 16weeks. As shown in Fig. 2A.i,B.i, physical activity as well as heat production of the SCH mice showed no differences compared with that of the controls. The AUC (area under the curve) for the two parameters (Fig. 2A.ii,B.ii) and the each corresponding average parameter in the light cycle and the dark cycle (Fig. 2A.iii,B.iii) also supported the conclusion. Consistent with the clinical traits of SCH, VO_2_ (oxygen consumption), VCO_2_ (production of carbon dioxide) and respiratory quotient (RQ, which is a measurement of the ratio between the O_2_ an organism intakes and the CO_2_ the organism eliminates and is expressed with the formula RQ = CO_2_ eliminated/O_2_ absorbed) were not different between the SCH mice and the control mice (Fig. 2C.i–E.i). The AUC results (Fig. 2C.ii–E.ii) and the corresponding average parameters (Fig. 2C.iii–E.iii) both respectively supported these conclusions, too. Unsurprisingly, BMR (the minimal rate of energy expenditure per unit time by endothermic animals at rest) and food intake were nearly identical as well (Fig. 2F).

These results suggested that the entire body metabolism showed no difference between SCH mice and control mice, which was consistent with the clinical features of SCH.

### Dyslipidemia and hepatic lipid metabolic disorders in SCH mice

**Figure 3:**
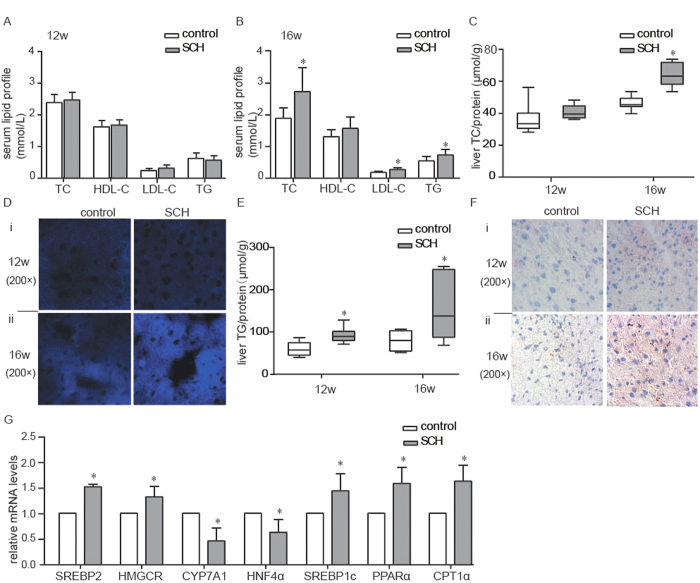
Hyperlipidemia and hepatic lipid metabolic disorders in the SCH mice. Male C57/BL6 mice were administered methimazole (MMI, 0.08 mg/kg·BW·d, SCH group, n = 6–8) or a corresponding volume of vehicle (control group, n = 6–8) for 12 weeks or 16 weeks. **(A)** Levels of total cholesterol (TC), high-density lipoprotein (HDL), low-density lipoprotein (LDL) and triglycerides (TG) in the serum of all mice were determined in the 12th week. **(B)** Levels of total cholesterol (TC), high-density lipoprotein (HDL), low-density lipoprotein (LDL) and triglycerides (TG) in the serum of all mice were determined in the 16th week. **(C)** Liver TC contents were measured using a cholesterol assay kit in the 12th week and the 16th week. **(D)** i, Filipin staining was used to measure liver cholesterol deposition in the 12th week. ii, Filipin staining was used to measure liver cholesterol deposition in the 16th week. Original magnification: 200×. **(E)** Liver TG contents were measured using a triglyceride assay kit in the 12th week and the 16th week. **(F)** i, Oil red O staining was used to measure liver TG accumulation in the 12th week. ii, Oil red O staining was used to measure liver TG accumulation in the 16th week. Original magnification: 200×. **(G)** Real-time PCR analysis of different lipid metabolism-related genes in the liver was performed in the 12th week. The data are presented as the mean ± SD. *p < 0.05 compared with the control group. The results are representative of 3 independent experiments.

SCH is often accompanied by dyslipidemia; thus, we first assessed the serum lipid profile in our SCH mouse model. However, in the 12th week, the mice begun to develop symptoms of SCH. Lipid levels of SCH mice were no different from those of control mice (Fig. 3A). In the 16th week, SCH had persisted for 4 weeks. Serum TC (total cholesterol), LDL-C (low-density lipoprotein-cholesterol) and TG (triglycerides) levels in SCH mice increased significantly relative to the controls, but there was no difference in serum HDL-C (Fig. 3B). These findings are consistent with previous studies^13^,^14^,^15^.

The liver plays a crucial role in lipid metabolism. Thus, we evaluated the hepatic lipid contents of control and SCH mice at 12 and 16 weeks in liver tissues.

As expected, the change in the hepatic TC contents in SCH mice was consistent with that of the serum TC levels. As shown in Fig. 3C, hepatic TC contents showed no significant differences between the SCH group and the control group in the 12th week. Notably, they were significantly elevated in SCH mice compared to that in the controls in the 16th week. Filipin staining is a classical technique that can be used to roughly quantify the cholesterol contents based on fluorescence intensity^19^. As shown in Fig. 3D, filipin staining was brighter in the livers of the SCH mice in the 16th week, which further confirmed the above results.

There was no difference in serum TG levels in the 12th week, but hepatic TG contents of the SCH mice increased significantly compared to those of the control mice, and this distinction was more obvious in the 16th week (Fig. 3E). Additionally, increased lipid droplet accumulation was observed in the livers of the SCH mice (Fig. 3F).

Moreover, to confirm the lipid metabolic disorders in SCH mice at the molecular level, we further measured the expressions of key molecules involved in hepatic lipid metabolism. HMGCR (3-hydroxy-3-methylglutaryl coenzyme A reductase) is the rate-limiting enzyme for cholesterol synthesis. CYP7A1 (cytochrome P450, family 7, subfamily A, polypeptide 1) is the rate-limiting enzyme for cholesterol conversion into bile acids. Interestingly, compared with the controls, HMGCR expression increased significantly while the expression of CYP7A1 decreased in the SCH mice (Fig. 3G). Therefore, the up-regulation of hepatic cholesterol synthesis and the reduction of hepatic cholesterol clearance ultimately contributed to the elevated cholesterol contents in the livers of the SCH mice. Additionally, expressions of key genes involved in TG synthesis, such as SREBP1C and PPARα, were significantly up-regulated, resulting in increased hepatic TG contents. (Fig. 3G). These results were consistent with our previous findings^20^,^21^,^22^.

Our results indicated that lipid metabolism in this SCH mouse model was highly disturbed. Hepatic TC and TG contents increased. Accordingly, serum TC and TG levels were also elevated.

### Hepatic ER stress was induced in SCH mice

The ER provides a specialized environment for the production and post-translational modification of lipids^23^,^24^,^25^. ER stress is involved in impairment of energy metabolism and inflammation, which is closely correlated with lipid metabolic disorders, such as diabetes mellitus, cardiovascular diseases, and NAFLD^26^,^27^,^28^. Whether ER stress is also involved in the lipid metabolic disorders in SCH is unknown. Thus, we first determined whether hepatic ER stress was induced in SCH mice.

Following ER stress, Bip (binding immunoglobulin protein) is displaced from the stress sensors IRE1α (inositol-requiring enzyme-1α), PERK (protein kinase RNA-like ER kinase) and ATF6α (activating transcription factor 6α) in the ER lumen, activating the three signaling pathways: the IRE1α/XBP-1 (X-box-binding protein-1) pathway, the PERK/eif2α (eukaryotic translation initiation factor 2α) pathway and the ATF6α pathway^29^.

**Figure 4:**
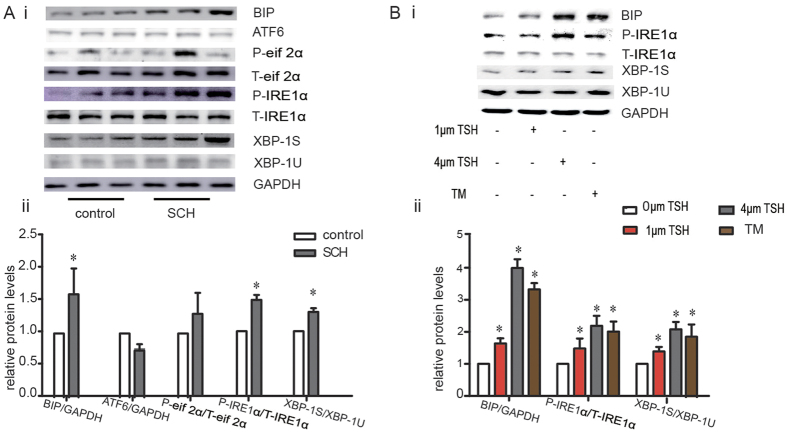
Elevated TSH triggered the liver ER stress response. **(A)** Male C57/BL6 mice were administered methimazole (MMI, 0.08 mg/kg·BW·d, SCH group, n = 6–8) or a corresponding volume of vehicle (control group, n = 6–8) for 12 weeks, and the expression of proteins in the ERS pathway was detected by western blot analysis (n = 6). The relative protein levels were quantified by densitometry and normalized to GAPDH or the corresponding total protein levels. **(B)** The Bip/IRE1α/XBP-1 pathway was analyzed in HepG2 cells treated with different doses of TSH (6 hours) using western blotting. Meanwhile, HepG2 cells were treated with TM (2.5 μg/ml) as a positive control, as it is a well-known agonist of ER stress. The relative protein levels were quantified by densitometry and normalized to GAPDH or the corresponding total protein levels. The data are presented as the mean ± SD. *p < 0.05 compared with the control group. The results are representative of 3 independent experiments.

Therefore, the expressions of all the key molecular indicators of ER stress were detected using immunoblotting. Compared to the controls, the SCH mice showed significantly up-regulated expression of Bip in the liver after administration of MMI for 12 weeks. Consistent with this result, the expressions of p-IRE1α (the active form of IRE1α) and XBP-1s (an active spliced form of XBP-1) in SCH mice were higher than those of the controls. However, the expression of ATF6α and p-eif2α did not show obvious differences in the two groups, which indicated that the ATF6α pathway and the PERK/eif2α pathway might not participate in the ER stress response in SCH mice (Fig. 4A).

To eliminate the influence of MMI and confirm the direct effect of increased TSH levels on ER stress, HepG2 cells were treated with different concentrations of bTSH. Additionally, TM (tunicamycin, an ER stress inducer) was applied as a positive control. Interestingly, after bTSH treatment, the protein levels of Bip, p-IRE1α and XBP-1s in HepG2 cells increased in a dose-dependent manner, which was consistent with the results observed *in vivo* (Fig. 4B).

The *in vivo* and *in vitro* results suggested that TSH could induce hepatic ER stress in the SCH mice, possibly via the IRE1α/XBP-1 pathway.

### ER stress may play an indispensable role in lipid metabolic disorders in SCH

Next, we determined whether hepatic ER stress played an indispensable role in the lipid metabolic disturbances accompanying SCH. SCH mouse model was established using the methods described above. After MMI was applied for 12 weeks, serum FT_3_, FT_4_ and TSH levels were measured to ensure the successful construction of the SCH model. After the SCH state was maintained for 2 weeks (i.e., MMI was used in drinking water for 14 weeks), we used 4-PBA (4-phenylbutyrate, a chemical chaperone that can inhibit ER stress in cells) to alleviate TSH-induced ER stress in SCH mice for 4 weeks to observe the serum lipid profile and liver lipid metabolism.

**Figure 5:**
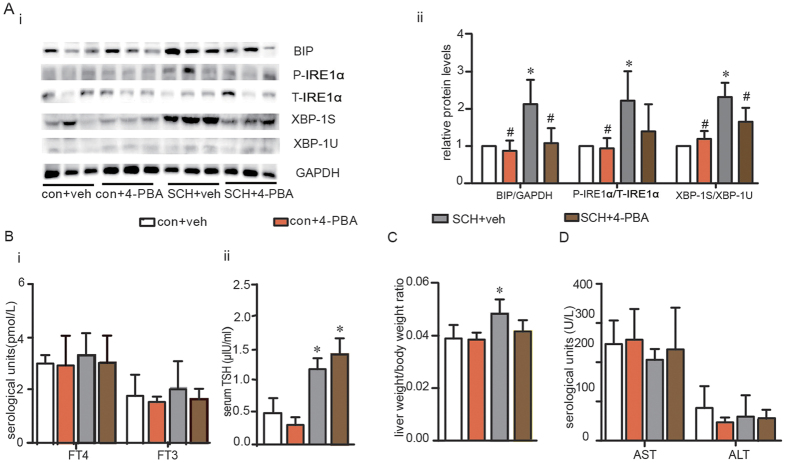
Treatment with 4-PBA alleviated TSH-triggered ER stress in liver. We established the SCH mouse model using MMI. After MMI was applied for 14 weeks, 4-PBA was administered by intraperitoneal injection at a dose of 100 mg/kg·BW·d for 4 weeks, and the mice were divided into four subgroups (as shown in Fig. S1): the vehicle-treated control mouse group (con + veh group), the 4-PBA-treated control mouse group (con + 4-PBA group), the vehicle-treated SCH mouse group (SCH + veh group) and the 4-PBA-treated SCH mouse group (SCH + 4-PBA group). **(A)** The Bip/IRE1α/XBP-1 pathway was analyzed in different groups by western blotting (n = 3). The relative protein levels were quantified by densitometry and normalized to GAPDH or the corresponding total protein levels. **(B)** Thyroid function was assessed(n = 6–12). **(C)** The ratio of liver weight to bodyweight was determined. (**D**) Liver function was measured (n = 6–12). The data are presented as the mean ± SD. *p < 0.05 compared with the vehicle-treated mouse group. ^#^p < 0.05 compared with the 4-PBA-treated SCH group. The results are representative of 3 independent experiments.

As shown in Fig. 5A, the liver ER stress response was assessed in four groups of mice. We found that the expressions of Bip, p-IRE1α and XBP-1s in 4-PBA-treated SCH mice were obviously down-regulated compared to those of the vehicle-treated SCH mice. Concurrently, 4-PBA injection for 4 weeks did not influence the mouse thyroid function (Fig. 5B). Compared with the vehicle-treated control mice, the liver weight to body weight ratio was substantially increased in vehicle-treated SCH mice; however, this ratio did not significantly change in 4-PBA-treated SCH mice (Fig. 5C). Additionally, 4-PBA has an outstanding safety profile *in vivo*^26^. As expected, it did not affect the liver function of the mice. (Fig. 5D).

**Figure 6:**
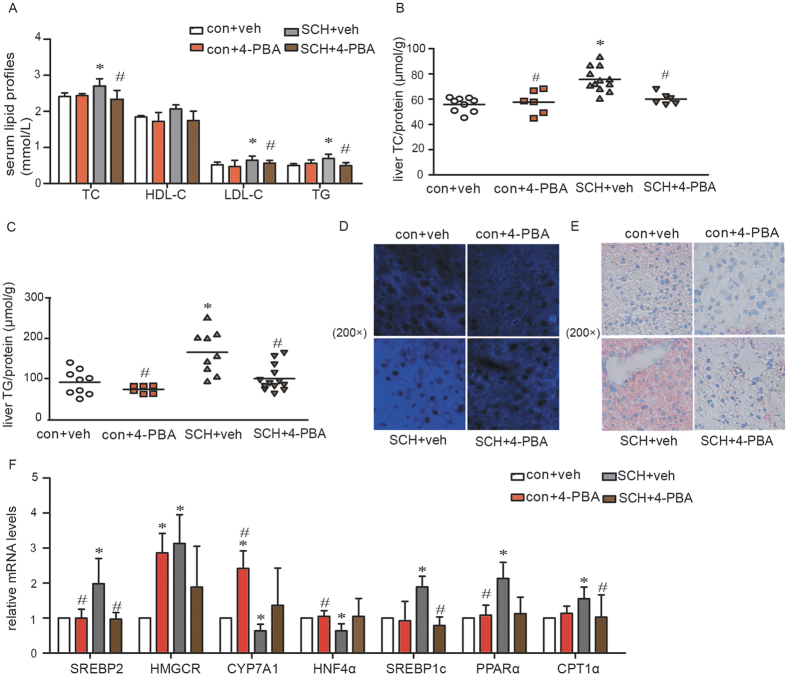
Treatment with 4-PBA alleviated lipid metabolic disorders in SCH mice. **(A)** Levels of triglycerides (TG), total cholesterol (TC), high-density lipoprotein (HDL) and low-density lipoprotein (LDL) in the serum of all mice were determined after 4-PBA was administered for 4 weeks (n = 6–12). **(B)** Hepatic TC contents were detected using a cholesterol assay kit after 4-PBA was administered for 4 weeks (n = 6–12). **(C)** Hepatic TG contents were detected using a triglyceride assay kit after 4-PBA was administered for 4 weeks (n = 6–13). **(D)** Filipin staining was used to measure hepatic cholesterol deposition after 4-PBA was administered for 4 weeks (n = 3). Original magnification: 200×. **(E)** Oil red O staining was used to measure hepatic TG accumulation after 4-PBA was administered for 4 weeks (n = 3). Original magnification: 200×. **(F)** Real-time PCR analysis of different lipid metabolism-related genes in the liver was performed in the 18th week (n = 4–6). The data are presented as the mean ± SD. *p < 0.05 compared with the vehicle-treated group. ^#^p < 0.05 compared with the 4-PBA-treated SCH group. The results are representative of 3 independent experiments.

Moreover, as shown in Fig. 6A, the serum lipids in the 4-PBA-treated SCH mice decreased into the normal range by inhibiting ER stress. We observed similar effects in the analysis of the liver lipid contents. Treatment with 4-PBA ameliorated the increase in hepatic TC (Fig. 6B) and TG contents (Fig. 6C) in SCH mice. Filipin staining (Fig. 6D) also supported the above findings. The frozen liver sections of vehicle-treated SCH mice exhibited the brightest fluorescence intensity. However, this phenomenon was strongly suppressed in SCH mice after 4-PBA treatment for 4 weeks. Consistent with these results, the liver fat decreases in 4-PBA-treated SCH mice were significantly reduced compared with vehicle-treated SCH mice as determined by Oil red O staining (Fig. 6E).

As shown in Fig. 6F, alleviating ER stress by 4-PBA strongly alleviated the expression of genes related to hepatic lipid metabolism. Interestingly, 4-PBA increased the expressions of both HMGCR and CYP7A1. When these two genes are both increased, they can offset their effect on cholesterol in the liver. Therefore, 4-PBA did not affect the hepatic cholesterol contents.

Thus, alleviating ER stress using 4-PBA in mice could significantly alleviate SCH-induced lipid metabolic disorders. Together, these findings demonstrate that hepatic ER stress played an important role in the lipid metabolic abnormalities in SCH.

## Discussion

Our findings demonstrated that a novel SCH mouse model could be established using MMI. Our data suggests that hepatic ER stress is induced in our new mouse model of SCH, which is associated with a phenotypic lipid disturbance. Although the exact mechanisms triggering the ER stress response in SCH were not fully elucidated, they likely involved the IRE1α/XBP-1 pathway, which exactly complemented the molecular mechanism of abnormal lipid metabolism in SCH. Interestingly, the enhancement in ER function induced by 4-PBA alleviating ER stress provides an unique approach to manage metabolic abnormalities associated with SCH.

As a commonly used and generally well-tolerated antithyroid agent^30^, MMI has been utilized to establish hypothyroidism animal models^31^ via its pharmacological mechanism [to prevent the synthesis of thyroxine (T_4_) and triiodothyronine (T_3_) by inhibiting the thyroid peroxidase]. In our study, we used an ultralow dose of MMI (which is equivalent to 1/10 to 1/15 of the minimum maintenance dose in adults) in mice to successfully construct the SCH mouse model. Compared with traditional modeling methods, such as hemi-thyroid electrocauterisation^11^ and thyroidectomy^12^ applied in rats, our method has the following features: (1) Modeling in different species. The use of a mouse model is an essential difference from the previous rat model. (2) Modeling using a noninvasive method. Using an antithyroid drug in the mouse model is more economic and easier, and above all, it will not subsequently produce unnecessary stress in mice without any trauma. (3) Effectively simulating the abnormal lipid metabolism of SCH. Several lines of evidence suggest that not only clinical hypothyroidism but also subclinical hypothyroidism is intimately associated with dyslipidemia and NAFLD^10^,^32^,^33^,^34^, which are the main clinical symptoms of lipid metabolism disorders. In our study, SCH mice were shown to have prominent dyslipidemia and liver fat depositions, vividly exhibiting the pathological characteristics of SCH.

The ambient temperature and adaptive thermogenesis are important factors in organism metabolism. In the metabolic chamber experiment, based on previous research^35^,^36^, we artificially controlled the ambient temperature at 24 °C, which was similar to the standard laboratory housing conditions (20 °C to 24 °C). Meanwhile, it also matched the room temperature (also between 20 °C to 24 °C) of the animal center. In our study, 24 °C is approximately equivalent to the basic temperature of mice in normal conditions, which can effectively avoid the influence of the ambient temperature variation on the metabolism. Adaptive thermogenesis is defined as non-shivering heat production in response to changes in environmental and physiological settings, such as cold, diet, fever, and stress^37^. Adaptive thermogenesis predominantly refers to non-shivering thermogenesis, which is principally mediated by BAT (brown adipose tissue)^38^. In our study, 24 °C is still below the thermoneutrality threshold for mice (30 °C). As a consequence, these animals likely have a degree of BAT activation and sympathetic signaling activated. Therefore, the adaptive thermogenesis could be active in both control and SCH mice.

Accumulating data have suggested that ER stress plays an important role in the development of dyslipidemia and NAFLD by aggravating inflammation, apoptosis, oxidative stress, insulin resistance and other factors^39^,^40^,^41^,^42^. However, in this study, we found that abnormal lipid metabolism was accompanied with ER stress mediated by elevated TSH. Then, what is the relationship between lipid metabolic disorders and ER stress in SCH? Next, we explored the role of ER stress in lipid metabolic disturbances in SCH. We used 4-PBA, a common chemical chaperone that inhibits ER stress in cells, in these studies as 4-PBA has an outstanding safety profile *in vivo*, it has been approved by the U.S. Food and Drug Administration, and it is broadly used in research of ER stress storage diseases, such T2DM^26^. After using 4-PBA as a chaperone for 4 weeks, ER stress in 4-PBA-treated SCH mice was obviously alleviated compared to vehicle-treated SCH mice. Our data further illustrated that TSH could trigger ER stress, which exacerbated lipotoxicity in SCH. Therefore, inhibiting ER stress would also improve SCH lipid metabolism. Thus, ER stress played an irreplaceable role in the lipid metabolic abnormalities in SCH.

IRE1α is the most conserved ER stress sensor and possesses both Ser/Thr kinase and endoribonuclease activity. IRE1α endoribonuclease activity splices the transcription factor XBP-1 (mRNA encoding X-box-binding protein) in mammals, producing an active spliced form of XBP-1s to activate the UPR pathway^43^. The up-regulations of Bip and XBP-1s are both key indicators of ER stress. In our experiment, we determined that the IRE1α/XBP-1 branch was the major pathway involved in stimulating ER stress, leading to the lipid metabolic disorders in SCH. The IRE1α/XBP-1 branch of ER stress has been implicated in lipid metabolism and is a crucial component in the maintenance of hepatic lipid metabolism under ER stress conditions. (1) The IRE1α/XBP-1 pathway is necessary for triglyceride biosynthesis. Mice with a specific deletion of IRE1α in hepatic cells showed altered expression of key metabolic molecules, for example, C/EBPβ, C/EBPδ, PPARγ and others, which are involved in triglyceride biosynthesis^44^. (2) This pathway is involved in the regulation of hepatic VLDL assembly and secretion, and it could increase microsomal triglyceride transfer protein activity^45^. (3) The IRE1α/XBP-1 branch of ER stress can regulate hepatic lipogenesis in hepatocytes by directly binding to the promoters of lipogenic genes, such as SCD1, DGTA2 and ACC2, to activate lipogenesis^46^. These findings not only extended the mechanism of abnormal lipid metabolism induced by elevated TSH but also suggested a new potential therapeutic target to prevent and treat lipid metabolic disorders in SCH.

There are still some limitations of our model: (1) The persistent status of SCH was limited with continuous administration of MMI in drinking water and lasted approximately 1.5–2 months. Therefore, this model is not suitable for experiments examining prolonged SCH. (2) Additionally, further studies on the specific mechanisms of ER stress induced by TSH in SCH should be carried out.

MMI can be used to establish a novel SCH mice model that shows similar pathological characteristics as SCH. ER stress may play a pivotal role in lipid metabolic disorders in the SCH mouse model. Additionally, inhibiting ER stress can ameliorate the lipid metabolic abnormalities in SCH. These findings suggest strategies for the prevention and treatment of lipid metabolic disorders and related diseases.

## Methods

### Animals and treatment

All mice were housed in an SPF room with controlled lighting (12 hours on, 12 hours off), and the temperature was maintained at 23 °C. Our research was approved by the Ethics Committee of Shandong Provincial Hospital (Jinan, China). The Shandong Provincial Hospital Animal Care and Use Committee approved the procedures for all animal experiments, and the methods were performed according to the approved guidelines.

### Generation of SCH mice

Male C57BL/6 mice (7 weeks old) were obtained from Vital River Corporation (Beijing, China). All mice were fed and allowed to acclimatize for one week. Then, the mice were divided into two groups: one group (MMI-treated group, n = 6–8) was administered MMI (0.08 mg/kg·BW·d), a drug that inhibits thyroid hormone synthesis, in the drinking water. The other group was provided with a corresponding volume of vehicle (control group, n = 6–8). The drinking water was estimated using a 10 ml graduated cylinder every 3 days. We weighed the mice every two weeks. Water consumption and the MMI dose were adjusted every 3 days based on the last drinking water and body weight. After MMI was administered for 12 weeks, 16 weeks and 20 weeks, the mice were fasted for 6 hours and then euthanized using pentobarbital sodium. Serum and liver samples were collected immediately prior to sacrificing the mice, and serum samples were tested for FT_3_ (free triiodothyronine), FT_4_ and TSH levels.

### Treatment of mice with 4-phenylbutyrate

We established the SCH mouse model using the methods described above. After MMI was applied for 12 weeks, serum FT_3_, FT_4_ and TSH levels were measured to ensure the successful construction of the SCH model. After the SCH state was maintained for 2 weeks (i.e., MMI was used in drinking water for 14 weeks), SCH mice were intraperitoneally injected with 4-phenylbutyrate (4-PBA, P21005, Sigma-Aldrich, St. Louis, MO, USA) in phosphate-buffered saline (PBS) or with vehicle. At the same time, control mice were intraperitoneally injected with 4-PBA or with vehicle. Then, 4-PBA was administered at a dose of 100 mg/kg·BW·d for 4 weeks. The specific protocols are shown in the flow chart (Fig. S1).

After MMI was administered for18 weeks, mice were fasted for 6 hours and were then euthanized using pentobarbital sodium. Serum and liver samples were collected and processed as described previously.

### Assessment of mouse body metabolism using metabolic chambers

While undergoing MMI treatment for 16 weeks, SCH mice (*n *= 3) and control mice (*n *= 3) were housed in individual metabolic chambers (PhenoMaster, TSE Systems, Germany) with a 12 hour dark-light cycle for 72 hours. The mice were maintained at 24 °C in this system. Mice acclimatized to the chambers for 24 hours following introduction, and baseline measurements began during the subsequent 48 hours. Food and water intake, physical activity, VCO_2_ (production of carbon dioxide), VO_2_ (oxygen consumption), RQ (respiratory quotient), heat production and BMR (basic metabolic rate)were recorded at 21 min intervals.

### Cell culture

The human hepatocellular carcinoma cell line (HepG2) was obtained from the Type Culture Collection of the Chinese Academy of Sciences, Shanghai, China. HepG2 cells were cultured in MEM/EBSS (HyClone, Logan, UT, USA) containing 10% fetal bovine serum (Gibco BRL, Gaithersburg, MD, USA), 100 units/ml penicillin, and 100 μg/ml streptomycin. Cells were incubated in a humidified atmosphere with 5% CO_2_ at 37 °C. When the cells reached 80% confluence, they were cultured in the presence of serum-free medium for 2 hours. Then, cells were treated with or without bTSH (bovine TSH, Sigma, St. Louis, MO, USA) or tunicamycin (TM, 2.5 μg/ml, Sigma-Aldrich, St. Louis, MO, USA) for 6 hours in serum-free medium.

### Thyroid function determination

Serum FT_3_ and FT_4_ levels were determined by competition radioimmunoassay (RIA) binding assays using a FT_3_ RIA Kit (Tian jin jiu ding, Tian jin, China) and a FT_4_ RIA Kit (Tian jin jiu ding, Tian jin, China) following the manufacturer’s instructions. Serum TSH was determined using a mouse ELISA kit (MyBioSource, San Diego, California, USA) following the product manual.

### Serum lipid profile and liver function assay

Serum triglycerides (TG), total cholesterol (TC), high-density lipoprotein (HDL), low-density lipoprotein (LDL), alanine transaminase (ALT) and aspartate transaminase (AST) in all mice were determined using an Olympus AU5400 automatic biochemical analyzer (Olympus Co., Ltd., Tokyo, Japan) in Shandong Provincial Hospital.

### Hepatic triglyceride and total cholesterol assays

The triglyceride content and total cholesterol in the liver were assayed using a triglyceride assay kit (E1015, Applygen Technologies, Beijing, China) and a total cholesterol assay kit (GPO-POD; Applygen Technologies, Beijing, China), respectively, according to the manufacturer’s recommended protocol.

### Oil red O staining

To determine hepatic lipid accumulation, the liver was carefully dissected and frozen in OCT embedding medium in liquid nitrogen. Frozen sections of the liver (5 μm) were stained with Oil red O for 10 min, washed, and counterstained with hematoxylin for 15 seconds. Representative photomicrographs were captured using a system incorporated in the microscope (Axiovert 100 M Zeiss, Zeppelinstrasse, Germany).

### Filipin staining

To measure hepatic cholesterol accumulation, the liver was carefully dissected and frozen in OCT embedding medium in liquid nitrogen. Frozen sections of the liver (5 μm) were stained using a filipin fluorescence staining kit (Genmed Scientifics Inc., USA), according to the manufacturer’s recommended protocol. All steps were performed under exclusion of direct light. These images were acquired by a fluorescence microscope (Axio Cam HRC, Zeiss, Germany) at 359/461 (excitation/emission) for filipin. And all images were exposured at the identical time: 120 ms.

### RNA extraction and real-time PCR

Total RNA was extracted from mouse livers using TRIzol. First-strand cDNA was generated using a commercial Prime Script RT reagent kit (TaKaRa, Otsu, Shiga, Japan). The resulting cDNA was amplified by real-time RT-PCR using SYBR Premix Ex Taq II (TaKaRa) and a LightCycler480 instrument (Roche Diagnostics). The mRNA levels were normalized to that of β-actin and presented relative to the control. The relative quantification of gene expression was performed using the 2^−ΔΔCt^ method^47^. The PCR primers are listed in Table 1.

### Western blot analysis

Liver and cells were lysed in RIPA buffer with a protease inhibitor cocktail, PMSF and sodium orthovanadate (Santa Cruz Biotechnology, Santa Cruz, CA). The protein concentration was quantified using a BCA protein assay (Pierce Biotechnology, Inc., Rockford, IL). Then, 80 μg of protein was resolved by SDS-PAGE, transferred to a membrane and blotted with specific antibodies: anti-phospho-IRE1α, anti-IRE1α (Abcam, Cambridge, MA), anti-XBP1 (Santa Cruz Biotechnology, CA), anti-phospho-eIF2α, anti-eIF2α (Cell Signaling Technology, Boston, MA), and anti-Bip (ProteinTech, Wuhan, China). The same membrane was stripped and reblotted with ananti-GAPDH antibody (Cwbiotech, Beijing, China) as a loading control.

### Statistical analysis

Data were analyzed using SPSS 17.0 and are expressed as the mean ± standard deviation. Differences between means were compared using either an unpaired Student’s t test for two-group comparisons or one-way analysis of variance (ANOVA) (Dunnett’s t test or LSD test) for multiple comparisons. Differences were considered significant at p < 0.05.

## Additional Information

**How to cite this article**: Zhou, L. *et al*. Endoplasmic Reticulum Stress May Play a Pivotal Role in Lipid Metabolic Disorders in a Novel Mouse Model of Subclinical Hypothyroidism. *Sci. Rep*. **6**, 31381; doi: 10.1038/srep31381 (2016).

## Supplementary Material

Supplementary Information

## Figures and Tables

**Table 1:** Quantitative RT-PCR primers.

Gene	Species	Forward primer	Reverse primer
β-actin	mouse	ACCCCAGCCATGTACGTAGC	GTGTGGGTGACCCCGTCTC
SREBP-2	mouse	ACCCCAGCCATGTACGTAGC	GTGTGGGTGACCCCGTCTC
CYP7A1	mouse	AGCAACTAAACAACCTGCCAGTACTA	GTCCGGATATTCAAGGATGCA
HNF-4α	mouse	GGATATGGCCGACTACAGCG	GCACCTTCAGATGGGGACG
SREBP-1c	mouse	GCGCTACCGGTCTTCTATCA	GGATGTAGTCGATGGCCTTG
PPARα	mouse	AAGGGCTTCTTTCGGCGAAC	TGACCTTGTTCATGTTGAAGTTCTTCA
CPT1α	mouse	TTGGGCCGGTTGCTGAT	GTCTCAGGGCTAGAGAACTTGGAA
