# Supplementary material for: Endoplasmic Reticulum Stress May Play a Pivotal Role in Lipid Metabolic Disorders in a Novel Mouse Model of Subclinical Hypothyroidism
Source: Sci Rep. 2016 Aug 19;6:31381. doi: 10.1038/srep31381 (PMC4990971; doi:10.1038/srep31381)
Supplement: Supplementary Information [file srep31381-s1.doc]

**Endoplasmic Reticulum Stress May Play a Pivotal Role in Lipid Metabolic Disorders in a Novel Mouse Model of Subclinical Hypothyroidism**

Lingyan Zhou^1,2,3^, Shuyan Ding^4^, Yujie Li^1,2,3^, Laicheng Wang^5^, Wenbin Chen^5^, Tao Bo^5^, Kunpeng Wu^1,2,3^, Congcong Li^6^, Xiaojing Liu^1,2,3^, Jiajun Zhao^1,2,3^, Chao Xu^1,2,3 *^ and Ling Gao^2,3,5*^

^*^ Corresponding authors: Dr. Chao Xu (doctorxuchao@163.com) and Dr. Ling Gao (gaoling1@medmail.com.cn)

**Affiliations:**

^1^Department of Endocrinology and Metabolism, Shandong Provincial Hospital affiliated to Shandong University, Jinan, Shandong, 250021, China,

^2^Institute of Endocrinology, Shandong Academy of Clinical Medicine, Jinan, Shandong, 250021, China,

^3^Shandong Clinical Medical Center of Endocrinology and Metabolism, Jinan, Shandong, 250021, China,

^4^Experimental Animal Center, Shandong Provincial Hospital affiliated to Shandong University, Jinan, Shandong, 250021, China,

^5^Scientific Center, Shandong Provincial Hospital affiliated to Shandong University, Jinan, Shandong, 250021, China,

^6^Jinan central hospital affiliated to Shandong University, Jinan, Shandong, 250021, China.

**Supplemental Experimental Procedures**

**Treatment of mice with 4-phenylbutyrate.** Male C57BL/6 mice (7 weeks old) were fed and allowed to acclimatize for one week. Then, the mice were divided into two groups: one group (SCH group) was administered MMI (0.08 mg/kg·BW·d), the other group was provided with a corresponding volume of vehicle (control group, ).

After MMI was applied for 12 weeks, serum FT_3_, FT_4_ and TSH levels were measured to ensure the successful construction of the SCH model. After the SCH state was maintained for 2 weeks (i.e., MMI was used in drinking water for 14 weeks), SCH mice were intraperitoneally injected with 4-phenylbutyrate (4-PBA) in phosphate-buffered saline (PBS) or with vehicle. At the same time, control mice were intraperitoneally injected with 4-PBA or with vehicle. Then, 4-PBA was administered at a dose of 100mg/kg·BW·d for 4 weeks. The specific protocols are shown in the flow chart (Fig.S1).

After MMI was administered for18 weeks, mice were fasted for 6 hours and were then euthanized using pentobarbital sodium. Serum and liver samples were collected and processed as described previously.

**Figure S1. Treatment of mice with 4-phenylbutyrate (4-PBA) in flow chart.**

**
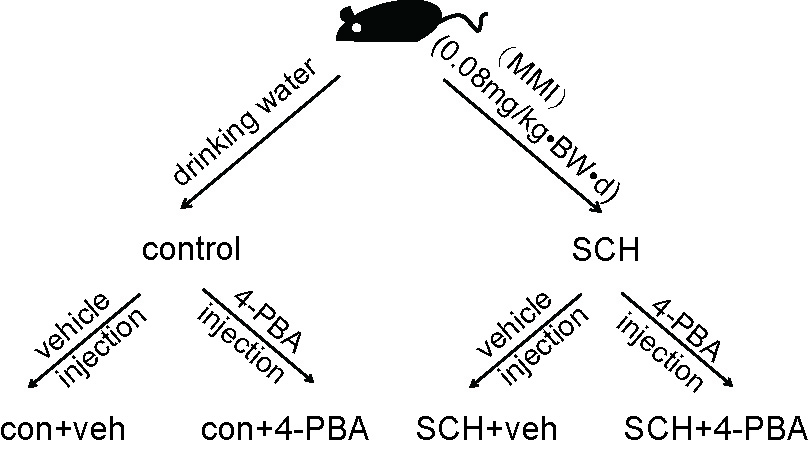
**
